# Trauma exposure and clinical presentation of UK veterans seeking specialist veteran mental health care

**DOI:** 10.1093/occmed/kqag039

**Published:** 2026-06-18

**Authors:** C Osório, L Culloty, S Talwar, S Ferrier, J Billings, T Greene

**Affiliations:** Division of Psychiatry, University College London, London, W1T 7NF, UK; Talking Therapies Southwark, South London and Maudsley NHS Foundation Trust, London, SE1 3SS, UK; Department of Language and Cognition, University College London, London, WC1N 1PF, UK; London Op COURAGE: Veterans’ Mental Health and Wellbeing Service, North London NHS Foundation Trust, London, NW1 0PE, UK; Division of Psychiatry, University College London, London, W1T 7NF, UK; Department of Psychology, Kingston University, Kingston upon Thames, KT1 2EE, UK; London Op COURAGE: Veterans’ Mental Health and Wellbeing Service, North London NHS Foundation Trust, London, NW1 0PE, UK; Division of Psychiatry, University College London, London, W1T 7NF, UK; Clinical, Educational and Health Psychology, University College London, London, WC1E 7HB, UK

## Abstract

**Background:**

Military veterans are at elevated risk of mental health conditions, including Post-Traumatic Stress Disorder (PTSD), Complex-PTSD (C-PTSD), depression and anxiety, among others. To address this need, in April 2023, the National Health Service (NHS) launched Op COURAGE, a specialized mental health service for veterans.

**Aims:**

To describe the demographics, military history, trauma-related factors and clinical presentation, including comorbidities, of UK help-seeking veterans.

**Methods:**

This retrospective study used data collected from a service evaluation involving veterans referred for support at an Op COURAGE service. Clinical data were gathered through self-report measures completed as part of routine clinical practice.

**Results:**

Clinical data were collected from 157 veterans referred to London Op COURAGE between April 2023 and March 2025 (out of a total population of 475). The majority were male (88%), with an average age of 49 years, and 83% served in the Army. A total of 48% experienced four or more adverse childhood experiences, and 55% experienced five or more lifetime traumatic experiences. Concerning mental health, 87% met criteria for depression, 79% for anxiety and 67% for PTSD/C-PTSD. Of the sample, 71% endorsed three or more mental health difficulties.

**Conclusions:**

Our findings indicate that help-seeking veterans present with a complex profile, characterized by high adversity and trauma exposure and significant mental health problems. Such complex mental health presentations can be difficult to treat, and these findings highlight the need for integrated care and tailored interventions. Future research could examine long-term outcomes of veterans with multiple comorbidities and investigate treatment adaptations.

Key learning points **What is already known about this subject:**

- Veterans face a heightened risk of developing mental health problems, including Post-Traumatic Stress Disorder (PTSD), Complex-PTSD (C-PTSD), depression, anxiety and substance misuse.
- Accessing suitable mental health support has historically been a challenge faced by many veterans.

**What this study adds:**

- Veterans assessed at London Op COURAGE exhibited a complex mental health profile, including 87% experiencing depression, 79% anxiety and 67% PTSD/C-PTSD.
- Additionally, 62% face emotional dysregulation, 57% anger, 54% suicidal or self-harming ideation, 34% alcohol misuse and 11% substance misuse; 71% reported three or more co-occurring mental health problems.

**What impact this may have on practice or policy:**

- The complex mental health profile of veterans emphasises the necessity for veteran-specific, multi-component and long-term treatment interventions.
- Further research on adapting treatment care for co-occurring mental health problems and assessing long-term outcomes to better support veterans.

## Introduction

Military veterans face a higher risk of developing serious mental health conditions, including Post-Traumatic Stress Disorder (PTSD), Complex-PTSD (C-PTSD), depression, anxiety and substance-related difficulties [1,2]. In a study of UK veterans resident in North West England, it was found that depression affected 18%, alcohol misuse 17%, anxiety 15%, PTSD 3% and substance misuse 1% [3], with many experiencing more than one mental health condition [3]. A separate study of UK veterans referred to a mental health crisis support service revealed that 68% experienced anxiety and depression, 67% had sleep disorders and 62% suffered from PTSD, with high comorbidity [1].

Veterans with a higher number of adverse childhood experiences (ACEs) are more likely to develop problems such as PTSD, depression, anxiety and aggression [4,5]. Moreover, during military service or deployment, exposure to combat and other potentially traumatic events can further compound these vulnerabilities [6,7]. Many studies have indicated that exposure to combat is associated with increased mental health problems, particularly PTSD, depression, anxiety, alcohol misuse and anger [7,8].

Former military personnel who have left the UK Armed Forces (UK AF) have unlimited access to National Health Service (NHS) services. Under the Armed Forces Covenant, UK veterans are entitled to priority treatment when their mental health problems are related to their military service [9]. However, veterans have often failed to engage with generic NHS mental health services, and when they do present, it is often with complex mental health challenges [10].

In April 2017, specialist NHS mental health services for UK veterans and reservists were set up across England. Between April 2017 and 2023, specialized assessments, treatment and onward referrals were provided by NHS Veterans’ Mental Health Transition, Intervention and Liaison Services (TILS); management of mental health crises was handled by the NHS Veterans’ Mental Health High Intensity Service (HIS); and intensive, complex mental health treatment was delivered through the NHS Veterans’ Mental Health Complex Treatment Services (CTS) [11]. In April 2023, these three NHS services were integrated into Op COURAGE to support veterans.

This study describes the demographic, military, trauma-related and clinical characteristics of UK help-seeking veterans referred to the London Op COURAGE service, with a focus on the complexity and comorbidity of their mental health presentations. We addressed the following research questions: First, what are the demographic and military service profiles and trauma histories of UK veterans seeking specialist mental health support? Second, what is the clinical presentation of mental health difficulties, including the extent to which comorbidity is present?

## Methods

Data for this study were obtained from the London Op COURAGE database, which routinely records veterans’ socio-demographic and clinical information at assessment. The database contained information on 475 veterans from all three branches of the UK AF (Army, Royal Air Force, Royal Navy) referred between 1 April 2023 and 31 March 2025. Of these, 157 veterans completed clinical measures. The discrepancy between the number of referrals and the number of participants completing the clinical measures was attributable to several factors, including logistical constraints, veterans’ refusals and staff not administering the measures. Eligibility for support included veterans who served in the UK AF and reside in Greater London.

This study was conducted as part of a service evaluation of London Op COURAGE, conducted by an external research team based at University College London (UCL). The research team received an anonymized database comprising data from veterans who either self-referred or were referred by external agencies. According to service protocols, all referrals were assessed between 5 and 10 working days. Subsequently, veterans underwent a comprehensive mental health assessment by a mental health professional (e.g. psychiatrist, clinical/counselling psychologist). Assessments covered mental health issues, risk, substance use and physical health problems. Afterwards, veterans received a treatment plan and were added to the waiting list. This study includes data from referral and initial assessment.

Socio-demographic information collected included sex, age, ethnicity, marital status, accommodation and employment status. Military characteristics included military branch, service length and year of discharge.

The ‘Life Events Checklist for DSM-5 (LEC-5)’ is a brief self-report screening tool assessing lifetime exposure to 17 potentially traumatic experiences, including natural disasters, combat and captivity. An additional item regarding exposure to bullying was added. The LEC-5 uses a six-point nominal scale that distinguishes between various exposures to an event, including direct witnessing or learning about it [12]. We only considered traumatic experiences that were directly experienced or witnessed by the veteran.

The ‘Adverse Childhood Experiences Questionnaire (ACE-Q)’ is a brief self-report screening tool assessing 10 potentially traumatic events, including abuse, neglect and household dysfunction during childhood/adolescence [13]. We defined a score of four or more as indicating significant adverse experiences during childhood/adolescence [13].

The ‘International Trauma Questionnaire (ITQ)’ is a self-report measure assessing PTSD and C-PTSD based on the International Classification of Diseases 11th revision (ICD-11) [14]. This tool evaluates key symptoms of PTSD, including re-experiencing, avoidance, hyperarousal and related functional impairments, as well as C-PTSD through Disturbances in Self-Organization (DSO), such as affective dysregulation, negative self-concept, disturbances in relationships and other related impairments [14]. Participants rate the frequency of each symptom experienced over the past month using a five-point Likert scale from 0 (‘not at all’) to 4 (‘extremely’). PTSD diagnosis is indicated by a score of two or more on at least one item within each PTSD symptom cluster, alongside evidence of functional impairment. C-PTSD diagnosis requires meeting PTSD criteria and additionally scoring two or more on at least one item in each DSO cluster, along with associated functional impairment [14].

The ‘Patient Health Questionnaire 9-Item (PHQ-9)’ is a self-report measure assessing symptoms of depression based on the Diagnostic and Statistical Manual of Mental Disorders, 5th Edition (DSM-5), including low mood, fatigue and suicidal/self-harming thoughts [15]. Participants rated each symptom over the past 2 weeks using a four-point Likert scale from 0 (‘not at all’) to 3 (‘nearly every day’). Scores of 10 or more indicate depression [15]. We independently analysed question nine of the PHQ-9, as it explicitly asks about thoughts of death and self-harm.

The ‘Generalised Anxiety Disorder 7-Item (GAD-7)’ is a self-report measure used to evaluate symptoms of anxiety based on the DSM-5, such as excessive worry and feeling afraid [16]. Participants rated each symptom over the past 2 weeks using a four-point Likert scale from 0 (‘not at all’) to 3 (‘nearly every day’). Scores of 10 or more indicate anxiety [16].

The ‘Alcohol Use Disorders Identification Test (AUDIT)’ is a self-report measure assessing key symptoms of alcohol-related issues, including harmful drinking patterns and hazardous alcohol dependence, developed by the World Health Organization (WHO) [17]. Participants rated their drinking using a four-point Likert scale from 0 to 4. Scores of eight or higher indicate significant alcohol-related harm [17].

The ‘Drug Use Disorders Identification Test (DUDIT)’ is a self-report measure assessing key symptoms of drug-related problems, including substance use and dependence, according to the ‘ICD-11’ and ‘DSM-5’ diagnostic criteria [18]. Participants rated each behaviour using a four-point Likert scale from 0 to 4. Scores of six or more in men and two or more in women suggest problematic drug usage [19]. Since our sample comprises 88% male veterans, we used the cut-off score of 6 for all participants.

The ‘Dimensions of Anger Reaction 5-Item (DAR-5)’ is a self-report measure assessing key symptoms of anger, including intensity, duration, frequency and impact on social functioning [20]. Participants rated each symptom over a 4-week period using a five-point Likert scale ranging from 1 (‘none or almost none of the time’) to 5 (‘all or almost all of the time’). Scores of 12 or higher indicate problematic anger issues [20].

The ‘Difficulties in Emotional Regulation 16-Item (DERS-16)’ is a self-report measure assessing key symptoms of emotional dysregulation, including negative emotional intensity, emotional impulsivity and psychological inflexibility [21]. Participants rated each symptom using a five-point Likert scale from 1 (‘almost never’) to 5 (‘almost always’). There is no consensus on the DERS-16 cut-off scores; however, we used a cut-off score of 50, as used by Moreta-Herrera *et al*. [22].

The ‘Work and Social Adjustment Scale (WSAS)’ is a self-report measure evaluating the impact of mental health on an individual’s life across five key areas, including work, home management, social activities, private activities and close relationships [23]. Participants rate how often their work and social life are affected using a nine-point Likert scale from 0 (‘not at all’) to 8 (‘very severely’). Scores of four or more on any question indicate some level of functional impairment, and a total score of 20 or higher suggests moderate functional impairment [23].

We received written confirmation from North London NHS Foundation Trust that this project was classified as a Service Evaluation, and no separate NHS ethical approval was required. The UCL research team managed the anonymized data in strict accordance with the research governance framework [24].

All analyses were conducted using Stata version 19.5 for Mac OS [25]. Descriptive statistics were used to summarize socio-demographic and military characteristics, adversity and trauma histories, and mental health presentations. To examine associations between categorical variables (e.g. mental health variables), we used chi-square (*χ*^2^) tests. Statistical significance was set at *P* < 0.05.

## Results

Of the 475 veterans referred to London Op COURAGE, all completed socio-demographic information; however, only 157 completed at least one clinical measure. Of the veterans who completed a clinical measure, the majority were male (88%, *n* = 138), with a mean age of 49 years (SD = 10.7, range 24–76), and a White ethnic background (75%, *n* = 117). Most served in the Army (83%, *n* = 130) and had a mean service length of 10 years (SD = 7.8, range 0–47 years) (Table 1).

**Table 1:** Socio-demographic and military characteristics of veterans

Socio-demographic		
	Full sample *n* = 434–475, *n* (%)	Sample completing ≥1 measure *n* = 141–157, *n* (%)
Sex		
Male	446 (94)	138 (88)
Female	29 (6)	19 (12)
Age		
Mean, SD,	47.9 (12)	48.7 (11)
Min–Max	22–90	24–76
21–40	150 (32)	41 (26)
41–60	248 (52)	94 (60)
≥61	77 (16)	22 (14)
Ethnicity		
White	346 (73)	117 (75)
Black	66 (14)	22 (14)
Asian	24 (5)	9 (6)
Mixed or multiple ethnic group	25 (5)	6 (4)
Missing data^a^	14 (3)	3 (2)
Marital status		
Single	195 (41)	59 (38)
Co-habiting or partnership	60 (13)	18 (12)
Married	82 (17)	37 (24)
Separated or divorced	46 (10)	14 (9)
Widowed	1 (0.2)	1 (0.6)
Missing data^a^	91 (19)	28 (18)
Accommodation		
Permanent	319 (68)	118 (75)
Temporary	85 (18)	25 (16)
Homeless	7 (2)	0 (0)
Missing data^a^	55 (12)	14 (9)
Employment status		
Unemployed	217 (46)	61 (39)
Full-time or part-time employment	142 (30)	66 (42)
Retired	31 (7)	7 (5)
Self-employed	27 (6)	7 (5)
Missing data^a^	53 (11)	16 (10)
Deprivation index		
Index 1 (most deprived area)	11 (3)	1 (1)
Index 2	86 (20)	30 (21)
Index 3	96 (22)	25 (18)
Index 4	52 (12)	12 (9)
Index 5	47 (11)	19 (14)
Index 6	30 (7)	8 (6)
Index 7	44 (10)	16 (11)
Index 8	33 (8)	17 (12)
Index 9	27 (6)	10 (7)
Index 10 (least deprived area)	8 (2)	3 (2)

Military service characteristics		
	Full sample *n* = 434–475, *n* (%)	Sample completing ≥1 measure *n* = 141–157, *n* (%)
Military branch		
Army	395 (84)	130 (83)
Royal Navy, including Royal Marine Commandos	43 (9)	16 (10)
Royal Air Force	26 (6)	9 (6)
Reserves (Army and Royal Air Force)	3 (1)	1 (1)
Missing data^a^	2 (1)	1 (1)
Length of military service	Mean, SD (8.7; 7.0) Min-Max (0–47)	Mean, SD (10.0; 7.8) Min–Max (0–47)
Year of discharge	Median (2008) IQR (1997–2014)	Median (2007) IQR (1997–2016)

Percentages were rounded to whole numbers.

a Participants who completed at least one measure. We selected depression because it had the highest completion rate.

SD, standard deviation; IQR, inter-quartile range.

The average score on the ACE was 3.4 (SD = 2.8, range 0–10). The most reported adversity faced by veterans during childhood included having a parent or adult swear at or humiliate them (57%, *n* = 78). Nearly half of veterans (48%, *n* = 66) experienced significant levels of childhood adversity, defined as a score of four or more ACEs (Table 2).

**Table 2:** Childhood and lifetime trauma exposure of veterans

Variables^a^			
Childhood adversity	Total (*N*)	Cases (*n*)	%
A parent or another adult in the household swore at you or humiliated you	136	78	57
A parent or another adult in the household often pushed, grabbed, slapped or threw you, and may have left physical marks	136	68	50
Your parents were separated or divorced	137	68	50
No one in your family showed love or cared for one another	137	54	39
A member of your household was depressed, had a mental illness or attempted suicide	135	47	35
You lived with someone who had problems with alcohol or drugs	135	43	32
You were often pushed, grabbed or slapped by your mother or stepmother	137	39	29
There was not enough food, you had to wear dirty clothes and no one was there to protect you	134	33	25
A member of your household was sent to prison	136	22	16
An adult or someone 5 years older than you touched your body in a sexual way or tried to have sex with you	135	21	16
**Lifetime trauma exposure**	**Total (*N*)**	**Cases (*n*)**	**%**
Bullying	85	60	71
Transportation accident (for example, car accident, boat accident, train wreck, plane crash)	94	57	61
Combat or exposure to a war-zone (in the military or as a civilian)	92	54	59
Any other very stressful event or experience	85	48	57
Assault with a weapon (for example, being shot, stabbed, threatened with a knife, gun, bomb)	89	48	54
Serious accident at work, home or during recreational activity	89	44	49
Fire or explosion	92	44	48
Life-threatening illness or injury	91	43	47
Severe human suffering	91	40	44
Sudden violent death (for example, homicide, suicide)	92	39	42
Sudden accidental death	92	31	34
Serious injury, harm or death you caused to someone else	90	30	33
Physical assault (for example, being attacked, hit, slapped, kicked, beaten up)	89	26	29
Other unwanted or uncomfortable sexual experience	88	18	21
Natural disaster (for example, flood, hurricane, tornado, earthquake)	90	18	20
Captivity (for example, being kidnapped, abducted, held hostage, prisoner of war)	79	15	19
Exposure to toxic substance (for example, dangerous chemicals, radiation)	92	16	17
Sexual assault (rape, attempted rape, made to perform any type of sexual act through force or threat of harm)	90	15	17

Percentages were rounded to whole numbers.

a The total number of responses varies according to each question. The LEC-5 only considered participants who ‘witnessed’ or ‘experienced’ each potentially traumatic event.

The average score on the LEC-5 was 5.3 (SD = 3.1, range 0–14). The most reported adversity experienced by veterans included bullying (71%, *n* = 60). More than half of veterans (55%, *n* = 53) experienced high levels of adversity, defined as endorsing five or more lifetime traumatic experiences (Table 2).

The prevalence of probable PTSD in the sample was relatively low at 6% (95% CI 2.7–11.5%), while a higher proportion, 61% (95% CI 52.0–69.3%), met the criteria for C-PTSD. The probable prevalence of depression and anxiety was also notably high, reported at 87% (95% CI 80.2–91.1%) and 79% (95% CI 71.3–84.3%), respectively. Additionally, over one-third (34%, 95% CI 26.4–42.0%) met the diagnostic criteria for alcohol misuse and 11% (95% CI 7.0–17.8) for probable drug misuse. Finally, veterans frequently reported difficulties with anger (57%, 95% CI 48.4–64.7%), emotional dysregulation (62%, 95% CI 51.6–71.7%) and suicidal or self-harming ideation (54%, 46.2–62.1%).

Overall, veterans demonstrated moderate to high levels of functional impairment across all assessed domains. The mean score was 21.9 (SD = 9.7, range 0–40). Veterans reported the most noticeable difficulties in social leisure activities (75%, 95% CI 67.3–81.7%) (Table 3).

**Table 3:** Mental health and psychosocial difficulties in veterans

Mental health difficulties						
Measure	Diagnosis	Mean value	Total sample	Positive cases^a^	Prevalence (%)	95% CI (%)
ITQ	PTSD	46.3	123	7	6	2.7–11.5
	C-PTSD			75	61	52.0–69.3
PHQ-9	Depression	16.6	157	136	87	80.2–91.1
	Suicidal ideation^b^	1.0	151	82	54	46.2–62.1
GAD-7	Anxiety	14.1	154	121	79	71.3–84.3
AUDIT	Alcohol misuse	7.1	142	48	34	26.4–42.0
DUDID	Drugs misuse	2.3	141	16	11	7.0–17.8
DAR-5	Anger	13.3	141	80	57	48.4–64.7
DERS-16	Emotional dysregulation	55.9	90	56	62	51.6–71.7

Psychosocial difficulties^c^					
Measure	Mean value	Standard deviation	Positive cases	Prevalence^d^ (%)	95% CI (%)
WSAS—social leisure activities	5.0	2.5	110	75	67.3–81.7
WSAS—close relationships	4.6	2.6	93	64	55.5–71.1
WSAS—home management	4.2	2.5	90	63	54.2–70.1
WSAS—private leisure activities	4.3	2.6	90	62	53.8–70.0
WSAS—work	4.5	2.8	71	61	52.0–69.7

Percentages were rounded to whole numbers. 95% CI were not rounded to avoid loss of precision.

a Positive cases reflect meeting the criteria for each measure.

b The PHQ-9 mean score includes response to Question 9, which assesses symptoms of suicidal and self-harming ideation. This question is asked separately to assess the risk of suicide or self-harming among veterans.

c WSAS was completed by 142 veterans.

d Differences in values are due to missing data in the questionnaires.

A total of 71% (*n* = 76) of veterans reported three or more mental health difficulties concurrently (mean = 3.2; SD = 1.4; range 0–6) (Table 4). Overall, we found a statistically significant association between depression and anxiety (*χ*^2^ (1) = 23.7, *P* < 0.001, 74%, *n* = 113), PTSD or C-PTSD and depression (*χ*^2^ (1) = 11.5, *P* < 0.001; 64%, *n* = 76) and PTSD or C-PTSD and anxiety (*χ*^2^(1) = 18.9, *P* < 0.001, 61%, *n* = 73) (Table 5).

**Table 4:** Comorbid mental health difficulties of veterans

Comorbid mental health difficulties	Positive cases	Prevalence (%)	95% CI (%)
0 mental health difficulties	4	4	1.4–9.6
1 mental health difficulty	10	9	5.1–16.6
2 mental health difficulties	17	16	10.1–24.2
3 mental health difficulties	28	26	18.6–35.4
4 mental health difficulties	28	26	18.6–35.4
5 mental health difficulties	16	15	9.3–23.1
6 mental health difficulties	4	4	1.4–9.6

These co-occurring comorbid mental health difficulties may include PTSD or CPTSD, depression, anxiety, alcohol use, drug use and suicidal ideation. Each disorder was made according to the measures criteria.

The total sample comprises 107 participants.

Percentages were rounded to whole numbers. 95% CI were not rounded to avoid loss of precision.

**Table 5:** Specific comorbid presentations

	PTSD and C-PTSD (*n* = 123)	Depression (*n* = 157)	Anxiety (*n* = 154)	Alcohol misuse (*n* = 142)	Drugs misuse (*n* = 141)	Suicidal ideation (*n* = 151)
PTSD and C-PTSD, *n* (%) (*χ*^2^)	–	76 (64) 11.5^***^	73 (61) 18.9^***^	28 (25) 0.21^ns^	7 (6) 0.49^ns^	48 (42) 12.47^***^
Depression, *n* (%) (*χ*^2^)	76 (64) 11.5^***^	–	113 (73) 23.66^***^	38 (27) 0.07^ns^	14 (10) 0.83ns	78 (52) 12.22^***^
Anxiety, *n* (%) (*χ*^2^)	73 (61) 18.9^***^	113 (73) 23.66^***^	–	35 (25) 0.02ns	13 (9) 0.81ns	71 (48) 14.49^***^
Alcohol misuse, *n* (%) (*χ*^2^)	28 (25) 0.21^ns^	38 (27) 0.07^ns^	35 (25) 0.02ns	–	10 (7) 8.11^**^	26 (20) 1.56ns
Drugs misuse, *n* (%) (*χ*^2^)	7 (6) 0.49^ns^	14 (10) 0.83ns	13 (9) 0.81ns	10 (7) 8.11^**^	–	11 (8) 4.10^*^
Suicidal ideation, *n* (%) (*χ*^2^)	48 (42) 12.47^***^	78 (52) 12.22^***^	71 (48) 14.49^***^	26 (20) 1.56ns	11 (8) 4.10^*^	–

The table displays the number of participants and prevalence rates. Please be aware that the total percentage exceeds 100% because each mental health condition was analysed independently, and the number of veterans (*n*) varies across conditions. Due to the low number of PTSD cases, PTSD and C-PTSD were combined into one variable.

ns, non-significant; *P < 0.05; **P < 0.01; ***P < 0.001.

Percentages were rounded to whole numbers.

## Discussion

Our findings highlighted the considerable mental health needs of this veteran sample, with a very high prevalence of depression, anxiety, and PTSD or C-PTSD, along with additional problems such as emotional dysregulation, anger, suicidal or self-harming ideation, alcohol misuse and substance misuse. There were high levels of comorbidity, with most of our sample experiencing three or more concurrent mental health difficulties. Our sample also had high levels of childhood adversity and lifetime exposure to traumatic experiences.

These findings are similar to those of two recent studies of UK veterans seeking psychological support. Campbell *et al*.’s [1] study included 180 veterans supported by the NHS Veterans HIS and reported a prevalence of depression and anxiety at 68%, PTSD at 62%, anger and emotional dysregulation at 48%, alcohol misuse at 43% and substance misuse at 32%. Notably, suicidal ideation was 85%, which is even higher than the 54% found in our study. Similarly, Williamson *et al*. (2023) studied 428 veterans supported by Combat Stress (a UK veterans’ mental health charity) and reported a prevalence of 81% for common mental disorders, 63% for C-PTSD and 56% for alcohol misuse. Additionally, 42% of veterans reported anger issues, 10% for substance misuse and 6.2% for PTSD [26].

Cumulatively, these studies, along with our findings, highlighted depression and anxiety as the main presenting difficulties among veterans, with an overall prevalence between 68% and 87% [1,26]. These figures are notably high, even compared to other studies of populations using specialized healthcare services. For example, a study of UK patients repeatedly referred to secondary care with medically unexplained symptoms found that around 48% met the criteria for depression, anxiety and panic disorder [27]. While it is well established that both depression and anxiety are among the most common reasons for individuals to seek health care [28, 29], the high prevalence within these veteran samples highlights the need to incorporate support for common mental health difficulties for military personnel, particularly on discharge from service.

Another notable finding was the extremely high prevalence of C-PTSD. Both our study and Williamson’s research indicated a prevalence between 61% and 63%. Research consistently shows that C-PTSD is more common in response to multiple or sustained exposure to traumatic events, including ACEs, prolonged domestic violence or exposure to combat or war zones [30]. Indeed, the veteran population in our study endorsed high levels of exposure to childhood adversity and lifetime trauma exposure. Notably, our study found significantly higher rates of childhood adversity than other UK studies with veterans seeking treatment [4,5]. Regarding lifetime exposure, our findings are comparable to those of Northern Ireland veterans with C-PTSD comorbidities [31]. Evidence regarding the impact of cumulative childhood and adult adversity is well-established, and, at the same time, it is important to note that the National Institute for Health and Care Excellence (NICE) guidelines in the UK are still being developed for C-PTSD [32]. The current findings underscore their urgency.

Our study showed that more than half of veterans experienced suicidal ideation in the 2 weeks up to their assessment. These findings are lower than those observed by Campbell *et al*.’s (2024), who reported a prevalence of 85%. Campbell’s study, however, involved veterans identified at ‘point of crisis’ by the NHS Veterans HIS, a service tasked with managing veterans experiencing a mental health crisis. Our study included veterans seeking specialist veteran mental health support, including urgent and intensive care. A prior study of UK veterans receiving support at Combat Stress found that unemployment and a history of childhood adversity were linked to increased suicidal ideation [33], and indeed many of the current sample were unemployed (46%) or had experienced high levels of childhood adversity (48%).

We also observed that more than half of the veterans in our study experienced difficulties with emotional regulation (62%) and anger (57%). Similar findings have been reported in other UK studies, although their prevalence was slightly lower [1,26]. Our findings, in combination with other studies, suggest that emotional dysregulation and anger are significant issues for veterans with PTSD or C-PTSD [34].

Our study identified a 34% prevalence of alcohol misuse and an 11% prevalence of substance misuse. These figures are lower than those reported in the two other studies involving UK veterans seeking psychological support, which documented a prevalence of 43–56% for alcohol misuse and 10–32% [1,26]. Veterans seeking help for mental health issues often report high levels of hazardous alcohol misuse and dependence [35]. Drinking as a way of coping with psychological distress is associated with higher alcohol use and dependence among adult populations [36].

Another notable finding from our research was the high prevalence of comorbidity among veterans. We found that 71% experienced three or more mental health conditions simultaneously, similar to previous findings [1]. The high rate of co-occurring mental health issues emphasises the complex presentation of veterans, indicating that standardized treatment approaches are likely to be insufficient to support these individuals. However, it is important to recognize that this study relates specifically to treatment-seeking veterans at a specialist veterans’ mental health service who have largely not been helped in their previous NHS care pathway. Indeed, studies involving a broader UK veteran population suggest a considerably lower prevalence of mental health problems. The most extensive longitudinal study of UK military personnel (including later-stage veterans) who served in Afghanistan (Operation HERRICK), Iraq (Operation TELIC) and those who were not deployed (Era group), followed participants on four separate occasions since June 2004 [37]. The findings from Phase 4 with veterans revealed a prevalence of 28% for common mental disorders (mainly depression and anxiety), 11% for PTSD, 9% for alcohol misuse and 7% for C-PTSD [37]. Lastly, a substantial proportion of veterans in our study are from the Army. These findings align with other UK veteran studies [1,4].

The strengths of this study include a robust and independent evaluation of a clinical service by a clinical academic team. Data analysed were collected using validated self-report clinical measures. Several limitations have also been identified. First, findings from a single Op COURAGE service in London may limit the extent to which these results could be generalized to other Op COURAGE or NHS services and outside of an urban or specialized care context. Second, of the 475 assessments completed, fewer than 33% (*n* = 157) of veterans completed at least some clinical measures, raising some questions about whether these data are representative of all the veterans assessed at the service. Third, collecting data on long-term physical health conditions (e.g. cardiovascular, respiratory and digestive conditions) would have given us a more comprehensive profile of UK veterans accessing the Op COURAGE service. Lastly, this study included a high proportion of army veterans, which may limit the generalization of findings to other non-army veteran populations.

This study showed that veterans undergoing an initial assessment at London Op COURAGE have a complex mental health profile. They reported high levels of lifetime traumatic exposure, along with a high prevalence of depression, anxiety and PTSD/C-PTSD. Most veterans had three or more concurrent mental health problems. Such complex mental health presentations could pose challenges for treatment, potentially requiring highly personalized, multi-component interventions delivered over long periods. Future research could explore long-term outcomes for veterans with multiple comorbidities and investigate treatment adaptations.
